# Steep Decline in Binding Capability of SARS-CoV-2 Omicron Variant (B.1.1.529) RBD to the Antibodies in Early COVID-19 Convalescent Sera and Inactivated Vaccine Sera

**DOI:** 10.3390/v14020335

**Published:** 2022-02-07

**Authors:** Wenhao Zhou, Ping He, Junhua Li, Huan Liu, Mengjuan Shi, Junping Yu, Hongping Wei

**Affiliations:** 1 CAS Key Laboratory of Special Pathogens and Biosafety, Center for Biosafety Mega-Science, Wuhan Institute of Virology, Chinese Academy of Sciences, Wuhan 430071, China; zyzwh1999@163.com (W.Z.); peace192@163.com (P.H.); Lijh@wh.iov.cn (J.L.); huanl88xiao@163.com (H.L.); shimengjuan20@mails.ucas.ac.cn (M.S.); 2 University of Chinese Academy of Sciences, Beijing 100049, China

**Keywords:** SARS-CoV-2, Omicron, variants of concern, antibody, receptor-binding domain, inactivated vaccine

## Abstract

A new SARS-CoV-2 variant B.1.1.529 was named by the WHO as Omicron and classified as a Variant of Concern (VOC) on 26 November 2021. Because this variant has more than 50 mutations, including 30 mutations on the spike, it has generated a lot of concerns on the potential impacts of the VOC on COVID-19. Here through ELISA assays using the recombinant RBD proteins with sequences the same to that of SARS-CoV-2 WIV04 (lineage B.1), the Delta variant and the Omicron variant as the coating antigens, the binding capabilities between the RBDs and the antibodies in COVID-19 convalescent sera and vaccine sera after two doses of the inactivated vaccine produced by Sinopharm WIBP are compared with each other. The results showed that the Omicron variant may evade antibodies induced by the ancestral strain and by the inactivated vaccine, with significant reduction in the binding capability of its RBD much greater than that of the Delta variant.

## 1. Introduction

A new variant of SARS-CoV-2 Omicron (B.1.1.529), which was reported in November 2021, was identified as variants under monitoring (VUM) on 24 November 2021, and designated as a variant of concern (VOC) by WHO two days later, while the Delta variant took one month from a variant of interest (VOI: 4 April 2021) to VOC (11 May 2021) (https://www.who.int/en/activities/tracking-SARS-CoV-2-variants, accessed on 21 Dec 2021). As of 21 December 2021, the Omicron variant has been reported in 106 countries across all six WHO regions (https://www.who.int/publications/m/item/weekly-epidemiological-update-on-covid-19, accessed on 21 December 2021). It was estimated that Omicron might spread faster than Delta, as indicated by WHO on 17 December 2021 (https://www.who.int/publications/m/item/enhancing-readiness-for-omicron-(b.1.1.529)-technical-brief-and-priority-actions-for-member-states, accessed on 17 December 2021). Omicron has more than 30-residue mutations in the spike protein in comparison to 15-residue mutations in the Delta variant [1], which might be the reason for its enhanced transmissibility [2]. The kinds of vaccines approved have played great roles in controlling the spread of SARS-CoV-2 [3,4]. However, with the evolution of SARS-CoV-2 and the emergence of VOCs, the antibodies generated by the current vaccines might lose neutralization capability to the variants [5,6,7]. Therefore, it is important to evaluate the capability of the current vaccines against different variants [8,9].

The gold standard for evaluating the efficacy of a vaccine is to determine the neutralization titers of immunized sera based on live-virus culture, such as the plaque reduction neutralization test (PRNT). However, the PRNT needs a biosafety level-3 laboratory and is costly and lengthy to perform. In order to rapidly estimate the neutralization activity of sera against newly emerged variants, assays based on RBD protein, which is the receptor-binding domain of the virus binding to host cells, can be used to estimate the neutralization activity to some extent [10].

Here through ELISA assays using the recombinant RBD proteins with sequences the same to that of SARS-CoV-2 WIV04 (lineage B.1), the Delta variant and the Omicron variant as the coating antigens, the binding capabilities between the RBDs and the antibodies in COVID-19 convalescent sera and vaccinated sera after two doses of the inactivated vaccine produced by Sinopharm WIBP are compared with each other. The results showed that the antibodies in both types of sera had slumped binding capability to Omicron RBD but small reduction of binding to Delta RBD compared with that to the B.1 RBD indicate a much higher capability of the Omicron variant to escape the inactivated vaccine and to infect previously recovered people than the Delta variant.

## 2. Materials and Methods

### 2.1. Samples

Eleven vaccine serum samples were collected from eleven healthy volunteers at about 3 weeks after a 2nd dose of the inactivated vaccine manufactured by Sinopharm WIBP (Wuhan Institute of Biological products Co., Wuhan, China). Seventeen serum samples of the convalescent patients infected by SARS-CoV-2 lineage B.1 were collected between February and May 2020 for neutralization tests. All sera were stored at −80 °C until use. The study has been approved by the ethical committee of Wuhan Institute of Virology, Chinese Academy of Sciences (No. WIVH17202102).

### 2.2. Plaque Reduction Neutralization Test (PRNT)

A total of 24 serum samples, including 7 inactivated vaccine-induced sera and 17 convalescent sera were used to evaluate the relationship between neutralization activity by PRNT and the binding capability of RBD to antibodies in the sera measured by ELISA. The PRNT is the same as a previous report [11] with slight changes. Briefly, Vero E6 cells were cultured in Dulbecco’s Modified Eagle Medium (DMEM) containing 10% fetal bovine serum (FBS; Biological Industries, 04–001–1ACS, Cromwell, CT, USA) and 100 U/mL of penicillin-streptomycin. The cells were seeded in 12-well plates and incubated at 37 °C with 5% CO_2_ until they formed monolayers. SARS-CoV-2 WIV04 solutions with titers of about 300 plaque-forming units (PFU) were mixed with four dilutions of each serum sample (20, 80, 320 and 1280 folds) and incubated for 1 h at 37 °C. Following this, the mixtures were transferred to 12-well plates containing Vero E6 cell monolayers, individually. After incubation at 37 °C for 1 h with 5% CO_2_, the mixture was removed from the wells and then the wells were overlaid with DMEM containing 2% FBS and 0.9% methyl cellulose and incubated at 37 °C with 5% CO_2_ for 4 days. Fixation was then conducted by adding 1 mL of 8% formaldehyde to each well and incubated for 20 min. Finally, the plates were stained with 0.5% crystal violet. PRNT50 (≥50% reduction in the number of the virus plaque) was used to show the neutralization titers, which were obtained according to the EC50 fitted by nonlinear regression with GraphPad Prism 7 software.

### 2.3. Enzyme-Linked Immunosorbent Assay (ELISA)

Three recombinant RBD proteins of SARS-CoV-2 B.1 strain (residues 319–537 of the SARS-CoV-2 spike protein) and variant B.1.1.529 (Omicron, residues 319–537 with 15 mutations of G339D, S371L, S373P, S372F, K417N, N440K, G446S, S477N, T478K, E484A, Q493R, G496S, Q498R, N501Y, Y505H) and B.1.617.2 (Delta, residues 319–537 with mutations of L452R and T478K) (Acro Biosystems, Beijing, China) were utilized for ELISA assay. Briefly, ELISA plates (Jet Biofil, Guangzhou, China) were coated with 100 μL/well of carbonate buffer (pH = 9.6) containing recombinant RBD proteins (5 μg/mL) and incubated at 4 °C overnight. Afterward, the plates were washed three times with PBS containing 0.05% Tween 20 (PBST), blocked with 330 μL/well of 5% skim milk powder (Oxoid, Altrincham, UK) in PBST for 2 h at 37 °C and then washed three times with PBST. Sera were diluted with PBST at 1:20 and 1:80 dilutions first and added to the coated wells (100 µL per well). The plates were incubated at 37 °C for 1 h and then washed thoroughly with PBST. Finally, horseradish peroxidase- (HRP) labeled rabbit antibodies against human IgG (Biodragon Immunotechnologies, Suzhou, China) at a dilution of 1:10,000 in PBST (100 µL/well) were added into the pates and incubated for 1 h at 37 °C. After washing with PBST, the plates were added with 100 µL/well of 3,3,5,5-Tetramethylbenzidine (TMB) (Tiangen Biotech, Beijing, China) and incubated for 10 min at room temperature. After the reaction was stopped by 2 M H_2_SO_4_, optical density (OD) was measured at 450 nm using a microplate reader (INFINITE 200 PRO, TECAN, Männedorf, Switzerland). Analysis was performed using GraphPad Prism 7 software.

## 3. Results

### 3.1. The Corresponding Relationship between RBD Binding Capabilities Measured by ELISA and the Neutralization of Sera

**Figure 1:**
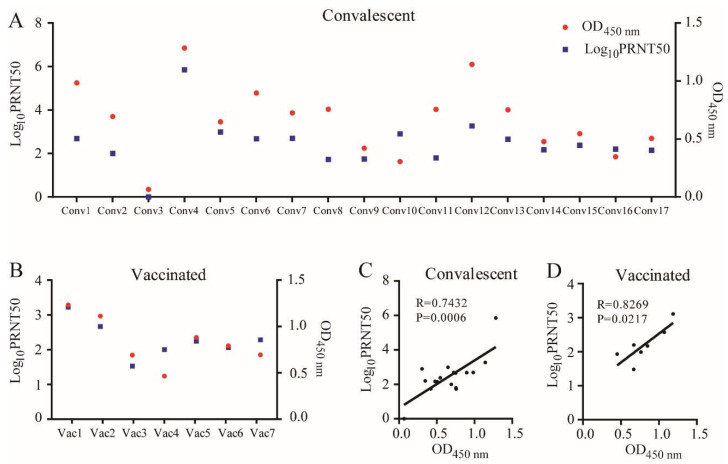
The correlation relationship between PRNT50 and OD_450__nm_ of ELISA (with sera dilution of 1:20). (**A**) PRNT50 (neutralization titers) of the seventeen convalescent sera and the corresponding OD_450__nm_ of ELISA; (**B**) PRNT50 of the seven vaccinated sera and the corresponding OD_450__nm_ of ELISA; (**C**) The correlation plots between the OD_450__nm_ and FRNT50 for the virus B.1. (**D**) The correlation plots between the OD_450__nm_ and PRNT50 for the virus B.1.

The PRNT50 values (the neutralization titers at 50% virus plaque reduction) for seven vaccinated sera and 17 convalescent sera against SARS-CoV-2 B.1 and the corresponding OD_450__nm_ measured by ELISA with the sera dilution of 1:20 using the RBD of SARS-CoV-2 B.1 as the coated antigen were listed in Table S1. The corresponding results were illustrated in Figure 1.

**Figure 2:**
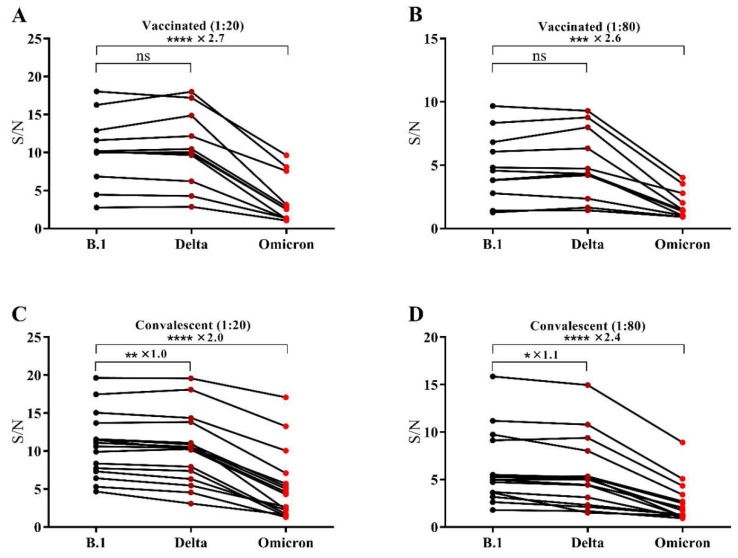
S/N values calculated from the data measured by a microplate reader at OD_450__nm_ of the vaccinated sera with the dilution of 1:20 (**A**) and with the dilution of 1:80 (**B**) and the convalescent sera with the dilution of 1:20 (**C**) and with the dilution of 1:80 (**D**). Data were paired-analysed by a one-way ANOVA with Dunn’s post-test; * *p* < 0.05, ** *p* < 0.01, *** *p* < 0.001, **** *p* < 0.0001, “ns” means “no significance”.

As shown in Figure 1A,B, for both vaccinated sera and convalescent sera, the PRNT50 values are generally correlated to the OD_450__nm_ values, indicating that higher OD_450__nm_ values were roughly corresponding to higher PRNT50 values, i.e., higher neutralization activity. Through Pearson correlation analysis (as shown in Figure 2C,D), a strong correlation between the PRNT50 values and OD_450__nm_ of RBD with sera for both convalescent group and the vaccinated group was evident. Therefore, measuring the changes in OD_450__nm_ by the ELISA method could provide a rapid indication for evaluating the changes in the neutralization activity of the sera. Because the Omicron variant was not available in our lab, the ELISA method was used to evaluate if Omicron might escape the antibodies induced by natural infections and by vaccination.

### 3.2. The Binding Capability of RBD from SARS-CoV-2 B.1, the Delta Variant and the Omicron Variant to Vaccinated Sera as well as Convalescent Sera

The S/N ratios of OD_450__nm_ of the vaccinated and convalescent sera to that of the antibody-negative control sera were used to express the binding capability of the RBDs to the antibodies in sera. S means signal, which is the OD_450 nm_ of the antibody-positive sera and and N means noise, which is the OD_450 nm_ of the antibody-negative serum. The results of 11 vaccinated sera were shown in Figure 2A,B. At both dilutions (1:20 and 1:80), antibodies in the vaccinated sera showed a small reduction (less than 20%) in binding to the Delta variant and a steep reduction (above 50%) in binding to the Omicron variant compared to that of B.1, as listed in Table S2. Similar results (Figure 2C,D) were also observed for the convalescent sera of the earlier B.1 strain (Table S3).

## 4. Discussions

In this study, the capability of the SARS-CoV-2 Omicron variant to escape the antibodies induced by inactivated vaccine and by natural infections of early variant was evaluated by the ELISA method using the corresponding RBDs as the coating antigens. Although the ELISA method is not equal to the classical neutralization assays based on cell culture, such as PRNTs, the OD_450__nm_ values were generally correlated to the PRNT50 of the serum (Figure 1), which is also observed by some previous reports [10] since the RBD of the SARS-CoV-2 spike protein, which can recognize ACE-2 on host cells, was proved to be the main receptor-binding domain to the host cell and was used to design the vaccines [12,13]. However, there are some limitations to using RBD binding capability to estimate the neutralization activity of the antibody in sera. Other fragments out of the RBD might neutralize the virus [14]. Furthermore, outside the RBD (RBD of Omicron contains 15 mutations compared to the lineage B.1 with reference sequence number of NC_045512.2 on NCBI), the Omicron variant has 15 mutations and several deletion or insertion sites, which might affect the conformation of the native RBD. As shown in Figure 1 and Table S1, there were two serum samples (Vac3 and Conv10) that did not show good correlation between the PRNT50 and the corresponding OD_450__nm_ values.

The Omicron variant has a total of 30 mutations on the spike protein, and half of the mutations happened on the RBD compared to the ancestral variant B.1, while there are eight mutations for the Delta variant, two of which exist on the RBD [15]. The results in Figure 2 revealed that the Delta variant, with only two mutations on the RBD, has limited decrease in the binding capability of the RBD to both vaccinated sera and convalescent sera, which was consistent to some studies recently [16,17]. However, Omicron evades antibodies elicited by the ancestral variants and by the inactivated vaccine efficiently, with steep reduction in the binding capability of its RBD.

Omicron is rapidly becoming dominant, and is replacing the Delta variant. The current results may indicate the need to develop boosters using the Omicron spike, rather than the ancestral or early variant spikes, for long-term boosting of immunity. This is especially pivotal when involving the induction of an adequate immune response in the elderly or the immunocompromised, who tend to respond weakly to the inactivated vaccine.

In conclusion, the initial results showed that Omicron may evade antibodies induced by the ancestral variants and by the inactivated vaccine efficiently, with reduction of more than 50% in the binding capability of its RBD much greater than that of the Delta variant.

## Data Availability

All available data are presented in this manuscript.

## Supplementary Materials

The following supporting information can be downloaded at: https://www.mdpi.com/article/10.3390/v14020335/s1. Table S1: The relationship between the neutralization titers of sera by PRNT50 and the corresponding RBD binding OD_450 nm_ by ELISA using RBD of SARS-CoV-2 B.1 as the coating antigen. Table S2: OD_450 nm_ of ELISA and the Omicron/Delta reduction rate of the RBD binding capability to the 11 vaccinated serum samples from ELISA results at dilutions of 1:20 and 1:80. Table S3: OD_450 nm_ of ELISA and the Omicron/Delta reduction rate of the RBD binding capability to the 17 convalescent serum samples from ELISA results at dilutions of 1:20 and 1:80.
