# Supplementary material for: Steep Decline in Binding Capability of SARS-CoV-2 Omicron Variant (B.1.1.529) RBD to the Antibodies in Early COVID-19 Convalescent Sera and Inactivated Vaccine Sera
Source: Viruses. 2022 Feb 7;14(2):335. doi: 10.3390/v14020335 (PMC8877760; doi:10.3390/v14020335)
Supplement: Supplementary file 1 [file viruses-14-00335-s001.zip › viruses-1548306-supplementary.pdf]

Supplementary materials

Steep Decline in Binding Capability of SARS-CoV-2 Omicron Variant (B.1.1.529) RBD to the Antibodies in Early COVID-19 Convalescent Sera and Inactivated Vaccine Sera

Wenhao Zhou [1,2,†], Ping He [1,2,†], Junhua Li [1,†], Huan Liu [1,2], Mengjuan Shi [1,2], Junping Yu [1,2,*] and Hongping Wei [1,2,*]

[1] CAS Key Laboratory of Special Pathogens and Biosafety, Center for Biosafety Mega-Science, Wuhan Institute of Virology, Chinese Academy of Sciences, Wuhan 430071, China; zyzwh1999@163.com (W.Z.); peace192@163.com (P.H.); Lijh@wh.iov.cn (J.L.); huanl88xiao@163.com (H.L.); shimengjuan20@mails.ucas.ac.cn (M.S.)

[2] University of Chinese Academy of Sciences, Beijing 100049, China

* Correspondence: yujp@wh.iov.cn (J.Y.); hpwei@wh.iov.cn (H.W.);
Tel.: +86-27-5186-1078 (J.Y.); +86-27-5186-1077 (H.W.)

† These authors contribute equally to this work.

Table S1. The relationship between the neutralization titers of sera by PRNT50 and the corresponding RBD binding $OD_{450\ nm}$ by ELISA using RBD of SARS-CoV-2 B.1 as the coating antigen. (Yellow items are with high PRNT50 and high QD_{450} with dilution 1:20. Green ones are the samples with low PRNT50 and low QD_{450}. Cyan ones are the samples with no relationship of PRNT50 and $OD_{450\ nm}$.

Sample	PRNT50/Dilution times	$OD_{450\ nm}$ (dilution 1:20)	$OD_{450\ nm}$ (dilution 1:80)
Vac1	>1280	1.1876	0.6373
Vac2	373	1.072	0.5494
Vac3	30	0.6686	0.2526
Vac4	86	0.4512	0.1836
Vac5	147	0.8509	0.4495
Vac6	98	0.7648	0.3176
Vac7	160	0.671	0.4001
Conv1	491	0.9844	0.5966
Conv2	101	0.694	0.344
Conv3	<20	0.0654	-
Conv4	>1280	1.2844	1.0373
Conv5	982	0.6485	0.3267
Conv6	478	0.8965	0.6374
Conv7	494	0.7255	0.3261
Conv8	54	0.7566	0.3093
Conv9	55	0.421	0.1712
Conv10	797	0.3053	0.2374
Conv11	63	0.7554	0.3606
Conv12	>1280	1.1431	0.7322
Conv13	451	0.7515	0.6627
Conv14	149	0.4786	0.2072
Conv15	241	0.5473	0.3228
Conv16	160	0.3469	0.1175
Conv17	142	0.5062	0.2411

Table S2. $OD_{450\ nm}$ of ELISA and the Omicron/Delta reduction rate of the RBD binding capability to the 11 vaccinated serum samples from ELISA results at dilutions of 1:20 and 1:80.

Sample name	1:20			1:20 (take the blank off)			Omicron reduction rate%*	Delta reduction rate%	1:80			1:80 (take the blank off)			Omicron reduction rate%	Delta reduction rate%
	Omicron	Delta	B.1	Omicron	Delta	B.1			Omicron	Delta	B.1	Omicron	Delta	B.1		
Vac1	0.5931	1.0685	1.1876	0.5399	1.0117	1.131	52.26	10.55	0.2475	0.5775	0.6373	0.1943	0.5207	0.5807	66.54	10.33
Vac2	0.499	1.1177	1.072	0.4458	1.0609	1.0154	56.10	-4.48**	0.2177	0.5446	0.5494	0.1645	0.4878	0.4928	66.62	1.01
Vac3	0.0768	0.5996	0.6686	0.0236	0.5428	0.612	96.14	11.31	0.063	0.2704	0.2526	0.0098	0.2136	0.196	95.00	-8.98
Vac4	0.0784	0.3861	0.4512	0.0252	0.3293	0.3946	93.61	16.55	0.0634	0.1466	0.1836	0.0102	0.0898	0.127	91.97	29.29
Vac5	0.1932	0.9234	0.8509	0.14	0.8666	0.7943	82.37	-9.10	0.1244	0.4963	0.4495	0.0712	0.4395	0.3929	81.88	-11.86
Vac6	0.4661	0.7548	0.7648	0.4129	0.698	0.7082	41.70	1.44	0.1713	0.2935	0.3176	0.1181	0.2367	0.261	54.75	9.31
Vac7	0.1763	0.6504	0.671	0.1231	0.5936	0.6144	79.96	3.39	0.091	0.3933	0.4001	0.0378	0.3365	0.3435	89.00	2.038
Vac8	0.1557	0.6116	0.6643	0.1025	0.5548	0.6077	83.13	8.70	0.0896	0.2682	0.3007	0.0364	0.2114	0.2441	85.09	13.40
Vac9	0.0651	0.1779	0.1821	0.0119	0.1211	0.1255	90.52	3.51	0.0567	0.0889	0.0926	0.0035	0.0321	0.036	90.28	10.83
Vac10	0.1557	0.6223	0.6572	0.1025	0.5655	0.6006	82.93	5.84	0.0836	0.2609	0.2511	0.0304	0.2041	0.1945	84.37	-4.94
Vac11	0.0839	0.2665	0.2938	0.0307	0.2097	0.2372	87.06	11.59	0.0566	0.1028	0.0835	0.0034	0.046	0.0269	87.36	-71.00

* The reduction rate is calculated as ($OD_{450\ nm}$ of B.1-$OD_{450\ nm}$ of the variant)/$OD_{450\ nm}$ of B.1*100%.

** The reduction rate of negative numbers means similar RBD binding capability of the serum sample to SARS-CoV-2 B.1.

Table S3. $OD_{450\,nm}$ of ELISA and the Omicron/Delta reduction rate of the RBD binding capability to the 17 convalescent serum samples from ELISA results at dilutions of 1:20 and 1:80.

Sample name	1:20			1:20(take the blank off)			Omicron reduction rate%*	Delta reduction rate%	1:80			1:80(take the blank off)			Omicron reduction rate%	Delta reduction rate%
	Omicron	Delta	B.1	Omicron	Delta	B.1			Omicron	Delta	B.1	Omicron	Delta	B.1		
Conv1	0.6288	0.9353	0.9844	0.5756	0.8785	0.9278	37.96	5.31	0.2711	0.6112	0.5966	0.2179	0.5544	0.54	59.65	-2.67
Conv2	0.3579	0.6791	0.694	0.3047	0.6223	0.6374	52.2	2.37	0.1695	0.3472	0.344	0.1163	0.2904	0.2874	59.53	-1.04
Conv3	0.0625	0.0651	0.0654	0.0093	0.0083	0.0088			0.0625	0.0651	0.0654	0.0093	0.0083	0.0088		
Conv4	1.0669	1.2736	1.2844	1.0137	1.2168	1.2278	17.44	0.90	0.5572	0.9734	1.0373	0.504	0.9166	0.9807	48.61	6.54
Conv5	0.2886	0.6688	0.6485	0.2354	0.612	0.5919	60.23	-3.40**	0.1121	0.3269	0.3267	0.0589	0.2701	0.2701	78.19	0
Conv6	0.4436	0.8995	0.8965	0.3904	0.8427	0.8399	53.52	-0.33	0.2129	0.5222	0.6374	0.1597	0.4654	0.5808	72.5	19.87
Conv7	0.3142	0.6869	0.7255	0.261	0.6301	0.6689	60.98	5.80	0.1289	0.2892	0.3261	0.0757	0.2324	0.2695	71.91	13.77
Conv8	0.1434	0.7208	0.7566	0.0902	0.664	0.7	87.11	5.14	0.0729	0.287	0.3093	0.0197	0.2302	0.2527	92.2	8.90
Conv9	0.1666	0.3554	0.421	0.1134	0.2986	0.3644	68.88	18.06	0.0827	0.1385	0.1712	0.0295	0.0817	0.1146	74.26	28.71
Conv10	0.1028	0.2015	0.3053	0.0496	0.1447	0.2487	80.06	41.82	0.0751	0.098	0.2374	0.0219	0.0412	0.1808	87.89	77.21
Conv11	0.3335	0.7081	0.7554	0.2803	0.6513	0.6988	59.89	6.80	0.1564	0.3414	0.3606	0.1032	0.2846	0.304	66.05	6.38
Conv12	0.829	1.1781	1.1431	0.7758	1.1213	1.0865	28.6	-3.20	0.3167	0.7027	0.7322	0.2635	0.6459	0.6756	61	4.40
Conv13	0.2683	0.6627	0.7515	0.2151	0.6059	0.6949	69.05	12.81	0.1086	0.3269	0.3451	0.0554	0.2701	0.2885	80.8	6.38
Conv14	0.1391	0.412	0.4786	0.0859	0.3552	0.422	79.64	15.83	0.0816	0.1518	0.2072	0.0284	0.095	0.1506	81.14	36.92
Conv15	0.1078	0.5168	0.5473	0.0546	0.46	0.4907	88.87	6.26	0.0693	0.3405	0.3228	0.0161	0.2837	0.2662	93.95	-6.57
Conv16	0.0816	0.2968	0.3469	0.0284	0.24	0.2903	90.22	17.33	0.0575	0.1093	0.1175	0.0043	0.0525	0.0609	92.94	13.79
Conv17	0.0887	0.4809	0.5062	0.0355	0.4241	0.4496	92.1	5.67	0.0643	0.2033	0.2411	0.0111	0.1465	0.1845	93.98	20.60

* The reduction rate is calculated as ($OD_{450\,nm}$ of B.1-$OD_{450\,nm}$ of the variant)/$OD_{450\,nm}$ of B.1*100%.

** The reduction rate of negative numbers means similar RBD binding capability of the serum sample to SARS-CoV-2 B.1.
